# Co-treatment of TGF-β3 and BMP7 is superior in stimulating chondrocyte redifferentiation in both hypoxia and normoxia compared to single treatments

**DOI:** 10.1038/s41598-018-27602-y

**Published:** 2018-07-06

**Authors:** Xiaobin Huang, Leilei Zhong, Janine N. Post, Marcel Karperien

**Affiliations:** Developmental BioEngineering, MIRA Institute for Biomedical Technology and Technical Medicine, University of Twente, Enschede, 7500 AE The Netherlands

## Abstract

Signaling by members of the transforming growth factor-β (TGF-β) superfamily, such as TGF-β3 and BMP7, and oxygen tension play a pivotal role in chondrocyte biology. The objective of this research was to investigate the endogenous BMP7 expression in human osteoarthritis (OA) cartilage and the effect of oxygen tension on the single or combined treatment with TGF-β3 and BMP7 on OA chondrocyte redifferentiation in three dimensional (3D) pellet cultures. The results showed the expression of BMP7 and its intracellular signaling target SMAD1/5/8 was decreased in early OA, while it was increased in later stages of OA. The combined treatment with TGF-β3 and BMP7, both in normoxia and hypoxia, was more effective than TGF-β3 or BMP7 alone in redifferentiating chondrocytes. This was reflected by Alcian blue/Safranin O staining and collagen type II protein expression, as well as by gene expression. Hypoxia elevated TGF-β3 and BMP7-induced matrix formation of OA chondrocytes and alleviated the catabolic gene expression. Interestingly, cells cultured under normoxia displayed mild signs of an inflammatory stress response, which was effectively counteracted by culturing the cells under low oxygen tension. Our data underscores the important modulatory role of oxygen tension on the chondrocyte’s responsiveness to TGF-β3 and/or BMP7.

## Introduction

Articular cartilage exhibits a poor self-regeneration capacity, and even minor cartilage defects may predispose to early onset osteoarthritis (OA). To break this vicious cycle, surgical intervention is often considered. Several surgical strategies have been developed to repair damaged cartilage. One of the most promising approaches is autologous chondrocyte implantation (ACI)^1^. This approach is based on isolation of chondrocytes, followed by expansion in monolayer culture to obtain sufficient cells for implantation into the lesion. However, monolayer expansion leads to a rapid loss of typical chondrocyte features, a process known as chondrocyte dedifferentiation, which eventually results in fibrous cartilage formation rather than hyaline cartilage after implantation^2,3^. Consequently, it is important to minimize the negative aspects of chondrocyte dedifferentiation or, alternatively develop effective strategies for chondrocyte redifferentiation.

Transforming Growth Factor-β (TGF-β) superfamily, including TGF-β and bone morphogenetic proteins (BMPs), is a family of secreted signaling factors with a remarkable ability to induce cartilage and bone formation. Generally, TGF-β induced signaling depends on Sma and Mad related proteins (SMAD) 2 and SMAD3^4,5^. This results in stabilization of the SOX9 transcription complex while inhibiting *RUNX2* expression^6,7^. BMP signaling depends on the SMAD1/5/8 pathway to stimulate the expression of typical cartilage hypertrophic markers, such as *COL10A1, MMP13* and *ALPL* during chondrogenesis of mesenchymal stem cells (MSCs) and termimal differentiation of primary chondrocytes^8^. TGF-β is an effective inducer of chondrogenesis^9,10^ by stimulating chondrocyte proliferation while inhibiting chondrocyte hypertrophy and maturation *in vitro*^11,12^. BMPs, such as BMP2 and BMP4, are potent regulators of chondrocyte hypertrophy and matrix degradation^13,14^. In contrast, BMP7 distinctly stimulates chondrocyte proliferation and inhibits chondrocyte hypertrophy^15–17^.

There are a few studies that have shown the combinationatorial effect of TGF-β and BMPs on chondrogenesis^18–20^. However, all these studies have been performed in normoxia (21% O_2_) which is a supra-physiological oxygen concentration compared to the much lower oxygen tension in articular cartilage *in vivo*. Articular cartilage is a typical avascular tissue, and chondrocytes normally reside in a low oxygen environment^21^. Hypoxia has a positive influence on the chondrocyte phenotype and has been shown to stimulate cartilage matrix formation^22,23^. Furthermore, most studies have focused on chondrogenic differentiation of MSCs from different sources with treatment of BMPs or TGF-βs. Few studies have focused on the redifferentiation of chondrocytes under hypoxic conditions in the presence of these factors. The aim of this study was to identify the effects of exogenous BMP7 and TGF-β3 on the redifferentiation of osteoarthritic chondrocytes after expansion in monolayer, using an aggregate pellet culture under low oxygen, and to explore the possibility of using the combination of hypoxia with TGFβ and BMP7 to stimulate chondrocyte redifferentiation.

## Materials and Methods

### Cartilage samples collection

The collection and use of human cartilage was approved by the local hospital ethical committee (De Medisch Ethische Toetsingscommissie (METC) TWENTE) and for all samples informed written consent was obtained. Cartilage specimens were isolated from 12 patients (mean age ± SD: 68 ± 6 years) with osteoarthritis undergoing total knee replacement surgery. In order to get comparable cartilage samples, several cartilage pieces were removed from the same joint and for each of the specimens a histological cartilage score was determined as previously described^24^.

### Cell culture and expansion

Human primary chondrocytes (hChs) were obtained from macroscopically healthy looking full thickness cartilage, dissected from knee biopsies of four OA patients (mean ± SD age 60 ± 3 years) undergoing total knee replacement, as described in^25,26^. hChs were used at passage 3 in this study.

### Pellet cultures and chondrogenic differentiation

Pellets consisting of 2.5 × 10^5^ cells were cultured for 3 weeks at 37 °C, 5% CO_2_, 21% O_2_ (for nomoxia) or 2.5% O_2_ (for hypoxia), in chondrogenic differentiation medium as described^27^. The medium was supplemented with 10 ng/mL TGF-β3 or 100 ng/ml BMP7 (R&D). The medium was refreshed twice per week.

### Total RNA extraction and quantitative polymerase chain reaction (qPCR)

Cell pellets were crushed and RNA was isolated using Trizol reagent (Thermo Fisher Scientific). The concentration and purity of RNA samples were determined using the Nanodrop 2000 (Thermo scientific). Total mRNA was reverse-transcribed into cDNA using the iScript cDNA Synthesis kit (Bio-Rad). qPCR was performed as described^28^.

### Alcian blue and Safranin O staining

Cartilage samples were fixed in 10% phosphate buffered formalin (pH = 7) overnight at room temperature (RT), dehydrated with a graded ethanol series and embedded in paraffin using routine procedures. Sections were cut at 5 μm thickness using a microtome (Shandon). Cell pellets samples were collected after 3 weeks of incubation and fixed with 10% buffered formalin. Sections of 7 μm thickness were directly cut using a cryotome (Shandon) after embedding in cryomatrix. Slides were stained as described^24,29^.

### Immunofluorescent (IF) staining for collagen type II and collagen type X

Immunofluorescent staining of collagen type II and collagen type X was performed using 7 μm sections from pellets. Slides were stained as described^26^ using Rabbit anti-human collagen type II antibody (ab34712, Abcam) or mouse anti-human collagen type X antibody (BIOCYC GmbH & Co. KG, Cat. No. 2031501005). Slides were viewed by BD pathway confocal microscopy.

### Immunohistochemistry (IHC) of BMP7 and SMAD 1/5/8 in cartilage or pellets

Immunohistochemistry staining of BMP7 and SMAD 1/5/8 for cartilage samples was performed using 5 μm sections, and the staining of SMAD 1/5/8 for pellet samples using 7 μm sections. The procedure was performed as described^24^. Mouse anti-human BMP7 (PeproTech) and mouse anti-human SMAD 1/5/8 antibody (Santa Cruz biotechnology) were used in this study. Images were taken using a Nanozoomer (Iwata City, Japan).

### EdU and TUNEL staining

For labeling of newly synthesized DNA in proliferating cells, EdU (5-ethynyl-2′-deoxyuridine) was added to the culture media at a concentration of 10 μM, 24 hours before harvesting the samples. Cell pellets were embedded in cryomatrix after fixing, and were cut into 7 μM sections by cryotome (Shandon). Sections were permeabilized and stained for EdU with Click-iT® EdU Imaging Kit (ThermoFisher scientific). Cryo-sections were also stained for DNA fragments with DeadEnd Fluorometric TUNEL System (Promega). Nuclei were counterstained with Hoechst 33342.

### GAG and DNA assays

After redifferentiation, pellets were digested and GAG was measured as previously described^30^. Relative cell number was determined by quantification of total DNA using a QuantiFluor® dsDNA System kit (Promega), according to the manufacturer’s instructions.

### Enzyme- linked immunosorbent assay (ELISA)

The supernatant of pellet cultures was collected every week. The content of MMP1 dissolved in medium was measured by ELISA using a mouse anti-human MMP1 antibody (MAB901-SP, R&D systems) as described^28^.

### Statistical analysis

Statistical differences between two groups were analyzed by two-tailed student’s t-tests or one-way ANOVA. P < 0.05 was considered statistically significant and indicated with an asterisk. Data are expressed as the mean ± SD.

## Results

### The expression of BMP7 and SMAD1/05/08 decreases in early OA while it increases in late stages of OA

**Figure 1:**
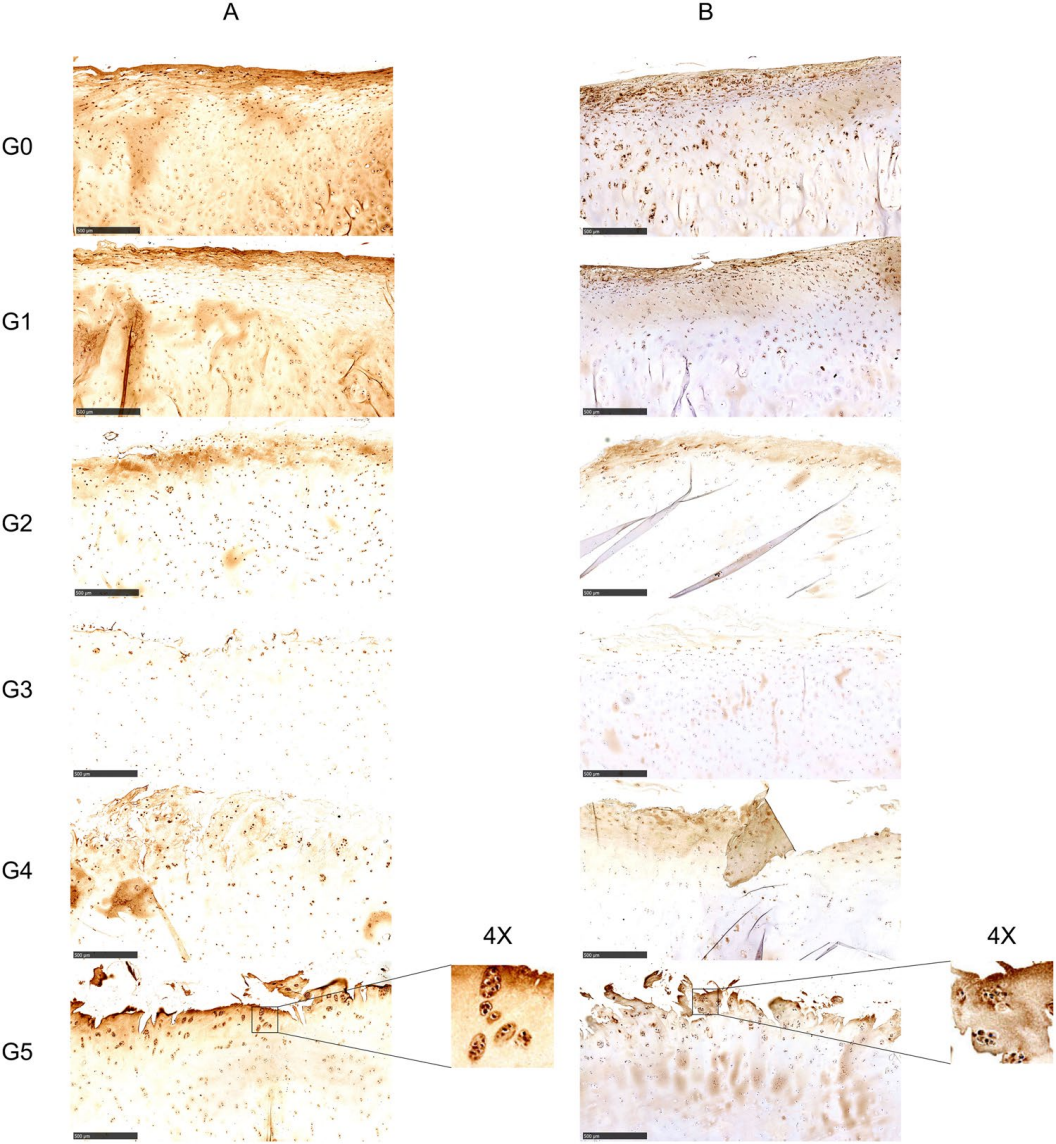
The protein expression of BMP7 and SMAD1/5/8 in human cartilage was visualized by IHC in each donor. The expression of BMP7 (**A**) and SMAD 1/5/8 (**B**) was visualized in differentially graded cartilage specimens of OA patients. Representative pictures are shown. Images were taken using the Nanozoomer (scale bar = 500 μm). G0, G1, G2, G3, G4, G5 = OARSI Grade 0, Grade 1, Grade 2, Grade 3, Grade 4, Grade 5. Magnified pictures were indicated in inserts.

To better characterize the BMP7/SMAD signaling pathway and its involvement in cartilage degeneration, we performed IHC to detect protein expression of BMP7 and its downstream target SMAD1/5/8 in OA cartilage. The expression of BMP7 (Fig. 1A) distinctly decreased with increasing severity from 0 to 3 in the early OA development, while it was increased from grade 3 to 5. BMP7 was mainly expressed in the matrix of the superficial layer and highly expressed inside chondrocytes. BMP7 expression was decreased in the middle layer and deep layers, however, it was increased again in hypertrophic chondrocytes. In the early OA stages from grade 0 to 2, positive staining of BMP7 was observed in the whole cartilage section, high to low from the superficial layer to the deep layer. At grade 3, intensity of BMP7 staining was decreased. BMP7 was only detected in chondrocytes in the superficial layer and in hypertrophic chondrocytes in the deep layer. Interestingly, in cartilage specimens with OA stage 4 and 5, and especially in grade 5 specimens, BMP7 was highly expressed in cell clusters, which is a typical characteristic of late OA. The expression of BMP7 was measured by IHC in each patient (Supplemental Fig. 1).

SMAD1/5/8 expression showed a similar trend as BMP7 (Fig. 1B). Its expression gradually declined from grade 0 to 3, and increased from grade 3 to 5. SMAD1/5/8 was mainly present in the chondrocytes of the superficial layer, even in the relatively healthy cartilage (grade 0 and 1). In stage 2 to 4 OA specimens, positive staining of SMAD1/5/8 was hard to observe in the chondrocytes of the middle layer and in hypertrophic chondrocytes of the deep layer. However, at stage 5, SMAD1/5/8 was strongly expressed in the top layer of the cartilage and especially in the chondrocyte clusters. The expression of SMAD1/5/8 was measured by IHC in each patient (Supplemental Fig. 2).

### Hypoxia enhanced matrix formation of OA chondrocytes with TGF-β3 or BMP7 treatment

**Figure 2:**
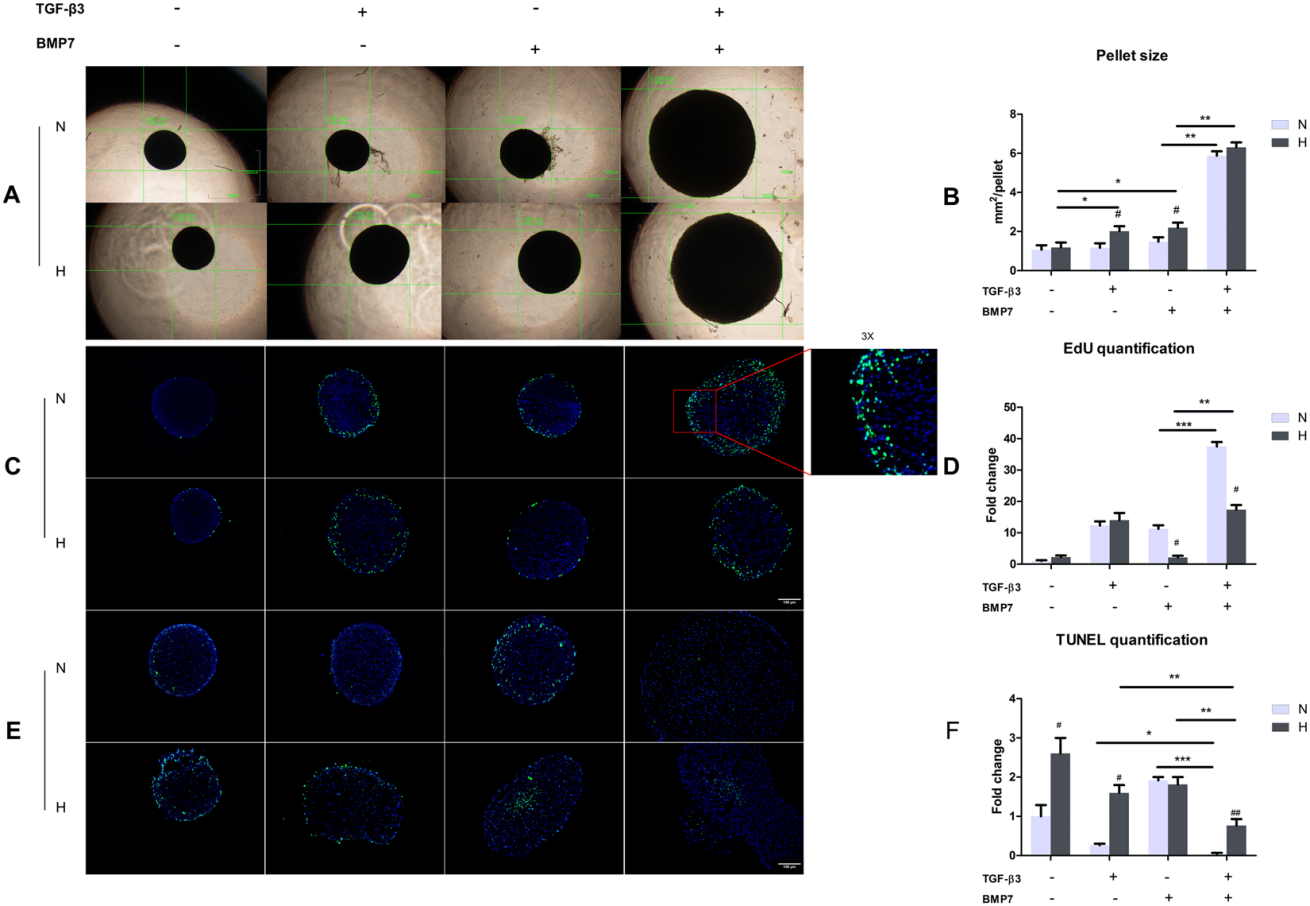
Cell proliferation and apoptosis in OA pellets. (**A**) Pellets imaged by light microscopy. Scale bar = 1000 μm. (**B**) Measurement of pellet size. Data represents the mean of three pellets in each group. ^#^Indicates a significant difference between normoxia and hypoxia; *represents P < 0.05; **represents P < 0.01; ***represents P < 0.001. Error bar represents Standard Deviation (S. D.). (**C**) EdU staining of pellets. EdU incorporation into newly synthesized DNA was visualized by Alexa 488 (green). Nuclei were counterstained with Hoechst 33342 (blue). Scale bar = 100 μm. (**D**) Quantification of EdU positive chondrocytes. (**E**) TUNEL staining of pellets. TUNEL positive cells were visualized with fluorescent labeling (green). Nuclei were counterstained with Hoechst 33342 (blue). Scale bar = 100 μm. (**F**) Quantification of TUNEL positive chondrocytes. N, normoxia; H, hypoxia; ^#^Indicates a significant difference between normoxia and hypoxia; *represents P < 0.05; **represents P < 0.01; ***represents P < 0.001. Error bar represents Standard Deviation (S. D.).

**Figure 3:**
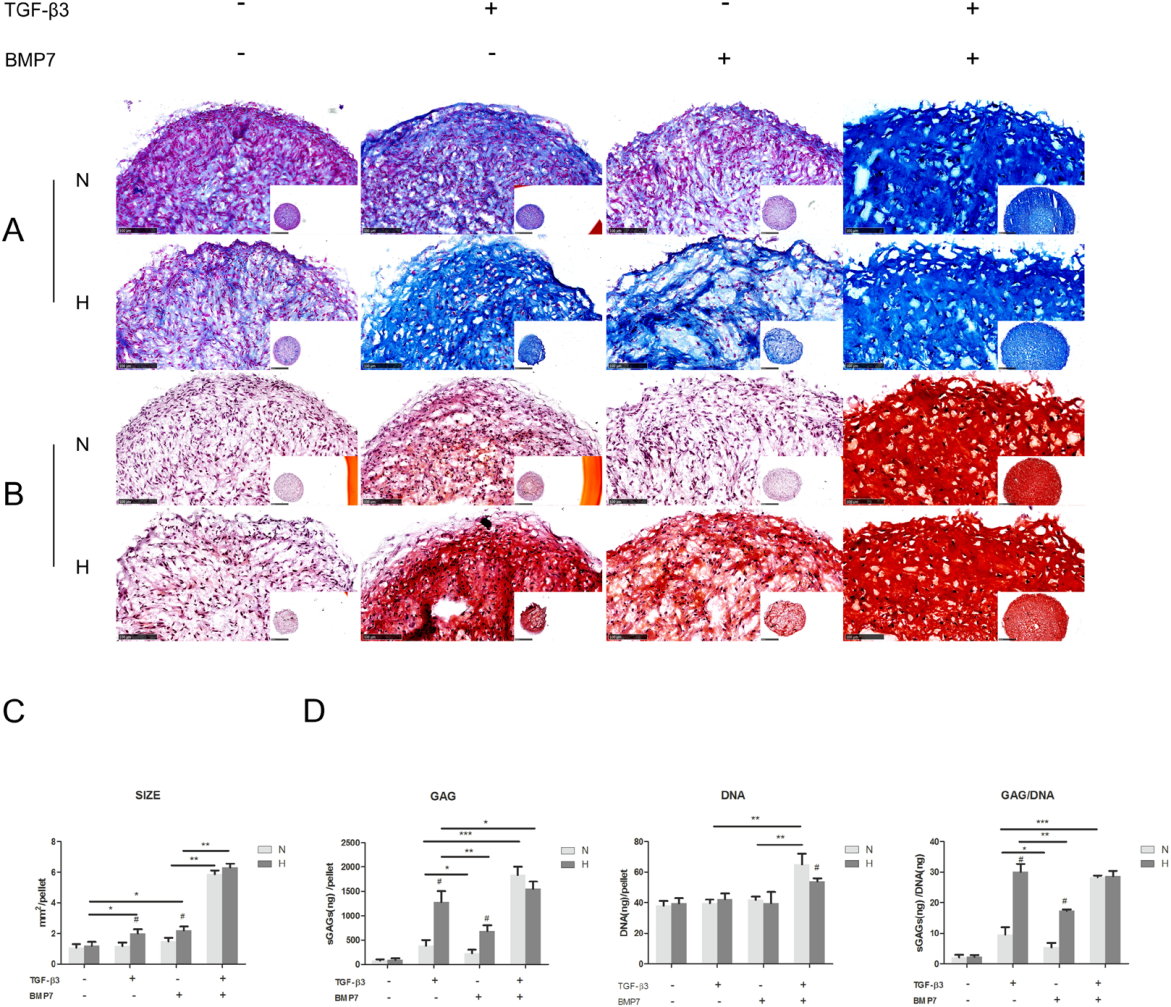
GAG production in pellets. (**A**) Alcian blue staining, nuclei were counterstained by nuclear fast red; (**B**) Safranin O staining, nuclei were counterstained by haematoxylin; (**C**) GAG and DNA assay. N, normoxia; H, hypoxia; *p < 0.05; **p < 0.01; ^#^represents a significant difference between normoxia and hypoxia (p < 0.05).

In 3D pellet cultures, the sizes of the pellets treated with either TGF-β3 or BMP7 under hypoxia were obviously larger than the pellets cultured under normoxia (Fig. 2A,B). There was no significant difference between normoxia and hypoxia in the TGF-β3 and BMP7 treated groups. To clarify whether the enhanced pellet size was due to cell proliferation or to matrix production, EdU proliferation assays, Alcian blue and Safranin O staining, and GAG assays were performed. As shown in Fig. 2C,D, the proliferation ratio after treatment with TGF-β3 did not change between normoxia and hypoxia, while BMP7 treated pellets under hypoxia presented less positive Edu staining than pellets in normoxia. Moreover, treatment of TGF-β3 or BMP7 pellets of hypoxia showed lower cell density than pellets cultured in normoxia, as can be derived from the DAPI staining. TUNEL assay showed that the amount of apoptotic cells was increased in hypoxia as compared to normoxia for TGF-β3 treatment and that no difference was observed for the BMP7 treated group (Fig. 2E,F). However, GAG deposition was increased in hypoxia after treatment with TGF-β3 or BMP7 (Fig. 3A,B). This was confirmed by GAG assays (Fig. 3C). Although we did not observe a difference in the amount of DNA between hypoxia and normoxia, GAG production was up to approximately 3 fold higher in hypoxia than in normoxia after treatment with TGF-β3 or BMP7. These observations indicated that the enlarged pellet size in hypoxia after treatment with TGF-β3 or BMP7 was mainly due to increased matrix production rather than increased cell proliferation.

### Synergistic effect of TGF-β3 and BMP7 on chondrocytes redifferentiation

As Fig. 2A,B shows, treatment with TGF-β3 or BMP7 alone did not significantly increase the pellet size in normoxia, which is in contrast to pellets treated in hypoxia. Interestingly, the combined treatment significantly increased the pellet size compared to the single treatments. The effect was more pronounced in normoxia than in hypoxia. EdU staining indicated that the treatment of TGF-β3 plus BMP7 greatly promoted cell proliferation in pellets in normoxia, while only a modest increase was found in hypoxia when compared to the TGF-β3 or BMP7 treated groups. In normoxia, BMP7 treated pellets showed more apoptotic cells. TGF-β3 increased the number of apoptotic cells in hypoxia as compared to nomoxia. Remarkably, co-treatment effectively decreased the number of apoptotic cells in both normoxia and hypoxia (Fig. 2E,F). Alcian blue (Fig. 3A) and safranin O staining (Fig. 3B) showed that TGF-β3 or BMP7 treatment dramatically enhanced GAG production in hypoxia, while this effect was not observed in normoxia. However, co-stimulation of TGF-β3 and BMP7 significantly increased GAG deposition both in normoxia and hypoxia. This similar trend was observed in the GAG assays, with the exception of the GAG/DNA content in the co-treatment with TGF-β3 plus BMP7 in hypoxia, which was not higher when compared to the treatment with TGF-ß3 alone in hypoxia. (Fig. 3C).

### Anabolic and catabolic gene expression profiles of OA chondrocytes treated with TGF-β3 and/or BMP7

**Figure 4:**
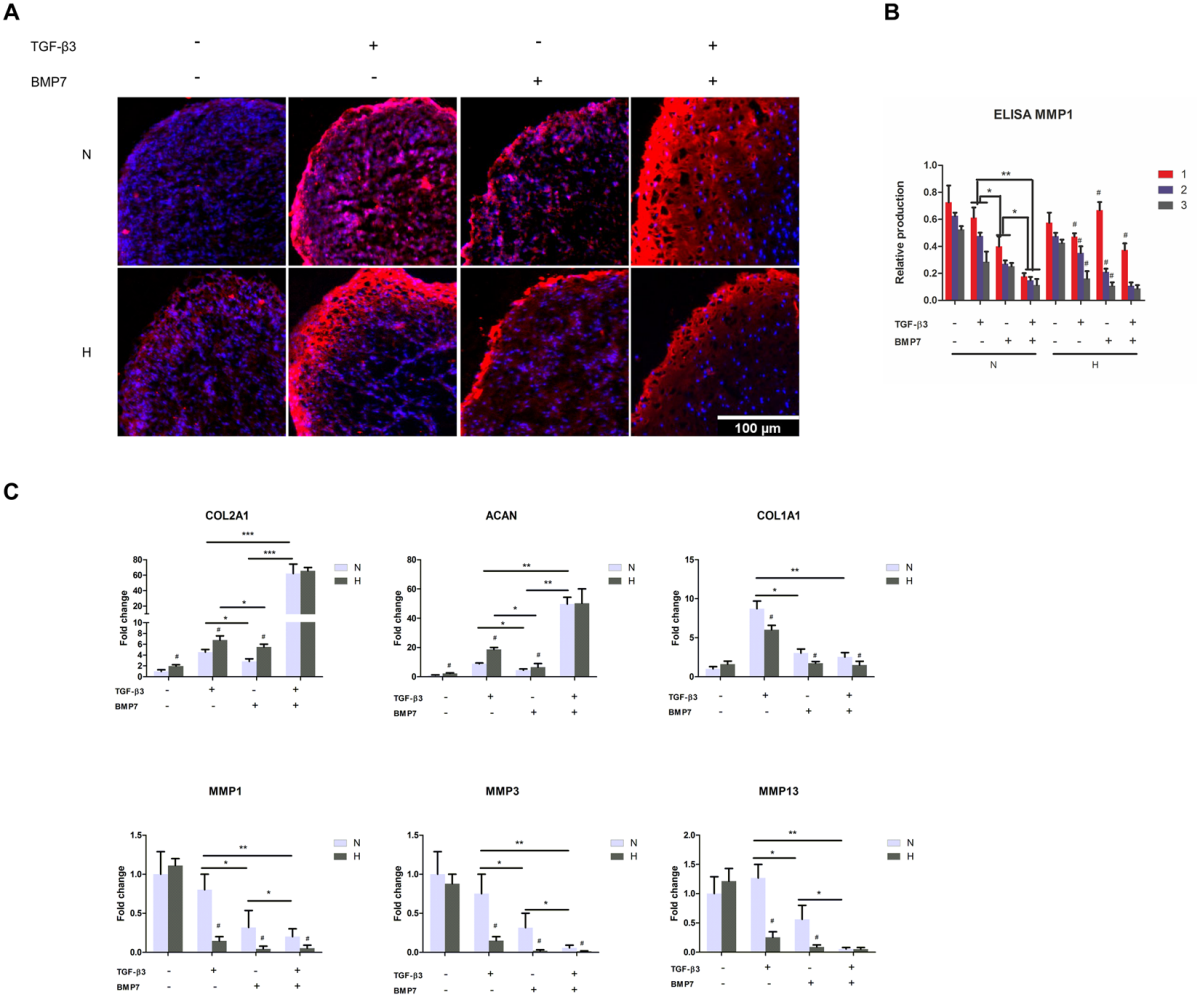
The anabolic and catabolic protein and gene expression profiles of OA chondrocytes after treatment with TGF-β3 and/or BMP7 in normoxia and hypoxia. (**A**) Measurement of type II collagen expression by immunofluorescence. Type II collagen was detected by rabbit anti-type II collagen antibody, followed by anti-rabbit secondary antibody coupled to Alex 564. Fluorescent images were taken by BD pathway confocal microscopy. Cell nuclei were counterstained with DAPI. Scale bar = 100 μm. (**B**) Secreted MMP1 was measured by ELISA. The numbers 1, 2, and 3 represent 1 week, 2 week, 3 week, respectively. (**C**) Gene expression of *ACAN, COL2A1, COL1A1, MMP1, 3, 13* was measured by Q-PCR. N, normoxia; H, hypoxia; *p < 0.05; **p < 0.01; ^#^indicates a significant difference with corresponding ones between normoxia and hypoxia (p < 0.05).

Next, we measured the expression and the distribution of type II collagen by immunofluorescence. Chondrocyte pellets treated with BMP7 or TGF-β3 alone increased the expression of type II collagen as compared to control (Fig. 4A) in normoxia. The most pronounced effect was found in the co-treatment group. In hypoxia, TGF-β3 was more effective in increasing the expression of type II collagen than BMP7. Co-treatment did not have an additive effect. The expression of collagen type X was also measured by immunofluorescence. As can be seen in Supplemental Fig. 3, TGF-β3 slightly induced the type-X collagen expression in normoxia only, BMP-7 alone did not induce collagen X expression. Both hypoxia as well as co-treatment with BMP-7 in normoxia effectively counteracted the induction of collagen X by TGF-β3.

To measure MMP1 secretion, medium was collected every week and MMP1 was measured by ELISA. The expression of MMP1 gradually decreased in the order of control, TGF-β3, BMP7, TGF-β3 + BMP7 in normoxia (Fig. 4). In hypoxia, the same trend was observed with the exception that MMP1 expression was relatively high in the first week as compared to in the pellets of this time-point in normoxia.

The anabolic and catabolic gene expression profiles were measured by RT-Q-PCR. The cartilage markers *ACAN* and *COL2A1* showed similar expression trends. Pellets with TGF-β3 treatment showed higher *ACAN* and *COL2A1* expression than BMP7 treatment. Co-treatment of BMP7 and TGF-β3 showed the highest expression of *ACAN* and *COL2A1* both in normoxia and hypoxia. The expression of *ACAN* and *COL2A1* is higher in hypoxia than in normoxia irrespective of the treatment with either TGF-β3 or BMP7. Catabolic genes, such as *MMP1, 3, 13* showed opposite trends compared to the anabolic genes. Their expression gradually decreased in the order of TGF-β3, BMP7, TGF-β3 + BMP7 both in normoxia and hypoxia. The combined group of BMP7 and TGF-β3 showed the lowest catabolic gene expression compared to single treatments. Catabolic gene expression in normoxia was higher than in the counter treatments cultured in hypoxia. This effect was less pronounced in the TGF-β3 and BMP7 co-treatment groups. The expression trend of the dedifferentiation marker *COL1A1* was similar to that of the catabolic genes, except for the negative control group (no TGF-β3 and no BMP7), which showed extremely low expression of *COL1A1*.

### SMAD 1/5/8 signaling in OA chondrocytes with treatment of TGF-β3 and BMP7

**Figure 5:**
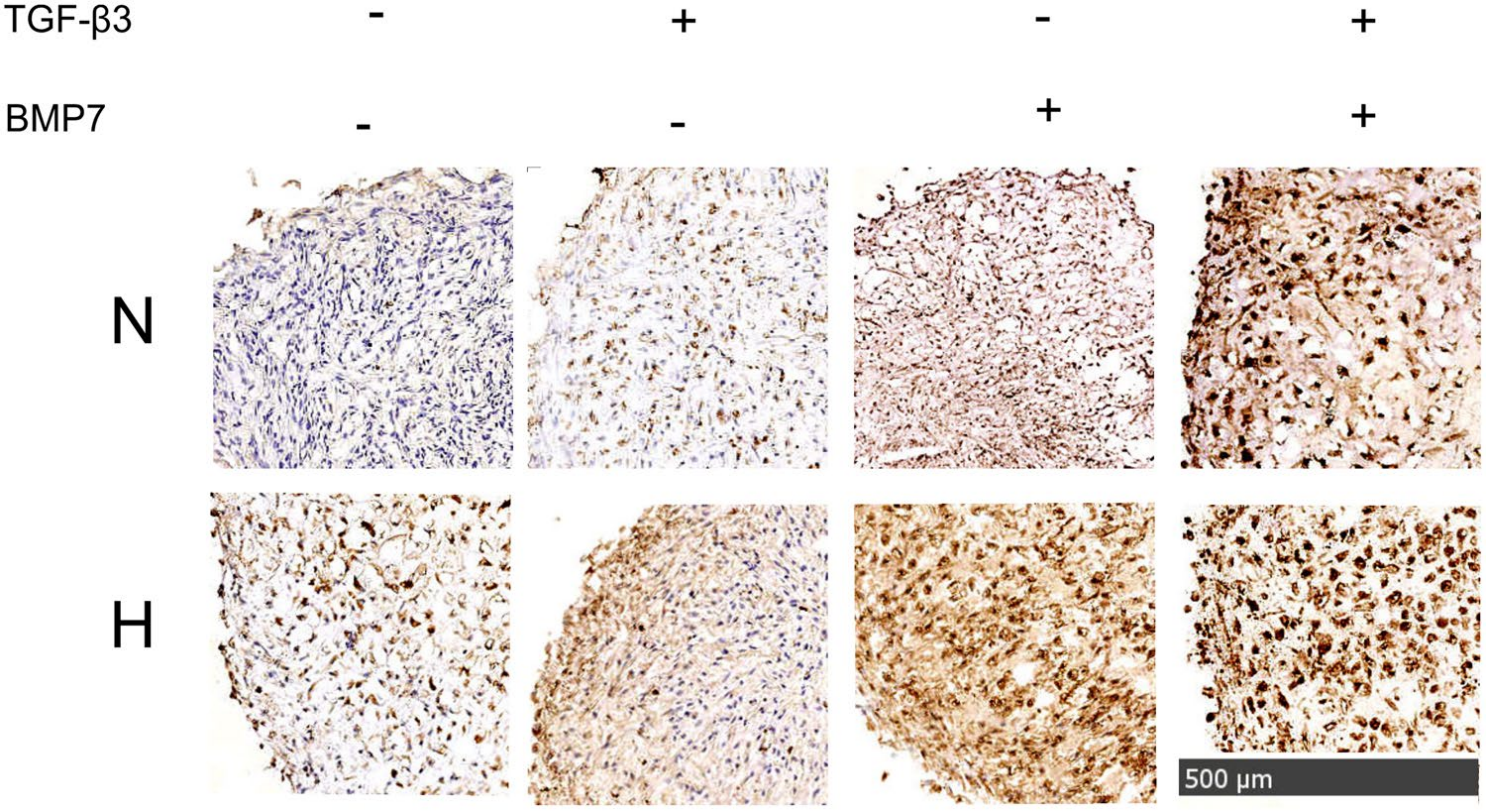
The expression of SMAD 1/5/8 in pellets in normoxia and hypoxia after treatment with TGF-β3 and BMP7. Protein expression of SMAD 1/5/8 was detected by mouse anti-SMAD 1/5/8 primary antibody, followed by a biotinylated anti-mouse secondary antibody followed by signal development using Horse Radish Peroxidase-Streptavidin. For visualization, DAB substrate kit was used. Images were taken by Nanozoomer. Nuclei were counterstained with haematoxylin. Scale bar = 500 μm. N, normoxia; H, hypoxia.

We next examined the translocation of SMAD 1/5/8 to the nucleus upon treatment with TGF-β3, BMP7 or in co-treatment in normoxia and hypoxia. The strongest positive staining was observed in the co-treatment group compared to the single treatments both in normoxia and hypoxia (Fig. 5). Stronger positive nuclear staining of SMAD 1/5/8 was observed in hypoxia than in normoxia.

## Discussion

Although intra-articular injection of BMP7 has been evaluated in clinical trials for the treatment of OA, only few studies have focused on the expression and role of endogenous BMP7 in human cartilage. To the best of our knowledge, this study has evaluated for the first time the protein expression of endogenous BMP7 and its intracellular signaling target SMAD 1/5/8 in different grades of OA in human cartilage. We show that BMP7 and SMAD1/5/8 are predominantly present inside cells and in the surrounding pericellular matrix of chondrocytes in the superficial and upper middle layers, and in chondrocytes of the transitional layer. Their expression initially decreased in early OA from grade 0 to 3 and then started to increase again from grade 3 to 5. At grade 5, BMP7 was especially highly expressed in chondrocyte clusters. Bobinac and his colleagues also found that BMP7 is strongly expressed in chondrocyte clusters^31^. Given the anabolic role of BMP7 in cartilage homeostasis, increased expression in chondrocyte clusters may be considered as a reparative response of the tissue. The expression of SMAD 1/5/8 followed the pattern of BMP7 expression albeit at a lower level.

Chondrocytes are the only cell type responsible for maintaining the surrounding matrix by synthesizing cartilage specific matrix proteins such as type II collagen and aggrecan^32^. Autologous Chondrocyte Implantation (ACI) has proven to be a pivotal approach as restorative cartilage surgery. However, even the most recent ACI techniques have some limitations^33^. It has been shown that the transplanted chondrocytes are able to form new cartilage, which appears to be of hyaline nature and expresses typical cartilage markers such as type II collagen, aggrecan and COMPs. However type I collagen and fibrous cartilage matrix are also detected in the newly formed tissue after two years^34,35^. There are plenty of cytokines and growth factors in the cartilage or synovial fluid to modulate the balance between the synthesis and degradation of ECM, or the balance between anabolism and catabolism. BMP/TGF-β signaling is extremely important in chondrocyte development. TGF-β induces collagen II and SOX9 deposition through SMAD2/3 phosphorylation pathway, while BMP7 promotes chondrocyte matrix production and inhibits chondrocyte hypertrophy via SMAD1/5/8 phosphorylation. The balance between SMAD 2/3 and SMAD 1/5/8 signaling, which are activated by TGF-β and BMP7 play a key role in cartilage homeostasis^36–38^. During ACI surgery, chondrocytes are isolated from a macroscopically healthy looking area in the joint, which may have limited matrix production capacity and the expansion process further reduces this matrix production potential. Therefore, to enable effective cartilage repair, efficient redifferentiation before or after implantation into the damaged area is a prerequisite.

BMP7 and TGF-β are important for the maintenance of cartilage homeostasis, and the loss of their expression could contribute to the development of OA. In this research, we mainly focus on the function of BMP7 and TGF-β in chondrocyte redifferentiation in 3D pellet cultures after expanding in monolayer. To do this, we cultured the pellets in serum free chondrogenic differentiation medium supplemented with TGF-β3 or BMP7 or both. *In vivo*, cartilage is exposed to a low oxygen tension. Despite this, chondrocytes are still cultured in normoxia in most labs. To study the effect of oxygen tension on the chondrocyte’s response to these growth factors, we compared the effects of growth factors on chondrocyte redifferentiation in both hypoxia (2.5% O_2_) and normoxia (21% O_2_). Our results indicated that TGF-β3 or BMP7 alone were not as efficient in redifferentiating chondrocytes as compared to the combined treatment with TGF-β3 and BMP7 both in normoxia and hypoxia. Alcian blue and Safranin O staining, GAG assay, type II collagen staining and gene expression analysis showed the same trend that the combined treatment with TGF-β3 and BMP7 positively influenced redifferentiation of OA chondrocytes. Previously, it was reported that BMP7 inhibited hMSC growth in expansion cultures^39^. We observed that the combination of BMP7 and TGF-β3 significantly promoted cell proliferation. However, the observed enlarged pellet size was mainly due to increased extracellular matrix production, with less tightly packed cells in the pellets.

In hypoxia, in the first week, MMP1 expression was relatively higher in the pellets treated with BMP7 alone or combined with TGF-β3, and lower in the TGF-β3 only treatment as compared to normoxia. This indicates that chondrocytes are more sensitive to the BMP7 stimulation in hypoxia in the early stages of redifferentiation. In this study, higher SMAD 1/5/8 expression was observed in hypoxia than in normoxia in all treatment conditions. While the expression of SMAD 1/5/8 was higher when BMP7 was present, the highest expression was observed in the combined treatment of BMP7 and TGF-β3 in hypoxia. Lafont and his colleagues reported that hypoxia inhibited BMP2 induced SMAD signaling in monolayer cultures of human articular chondrocytes^40^. These observations indicate that hypoxia modulates the sensitivity of the chondrocytes for BMP signaling.

The mechanism of the additive effects of BMP7 and TGF-β3 on chondrocyte redifferentiation and the influence of oxygen tension on this mechanism is still not clear. It might be that TGF-β3 upregulates the expression of BMP receptors in chondrocytes, which in turn promotes the cellular response to BMP7. It has been reported that TGF-β3 mediates the upregulation of the type I BMP receptor, ALK6, through which BMP7 and other BMPs are recruited to stimulate signal transduction in human MSCs^41^. Furthermore, it has been reported that the TGF-β3 receptor can also function as a BMP receptor by binding multiple members of the BMP subfamily including BMP7^42^. The mutual regulation or receptor activation may amplify and enhance the effects of TGF-β3 and BMP7.

**Figure 6:**
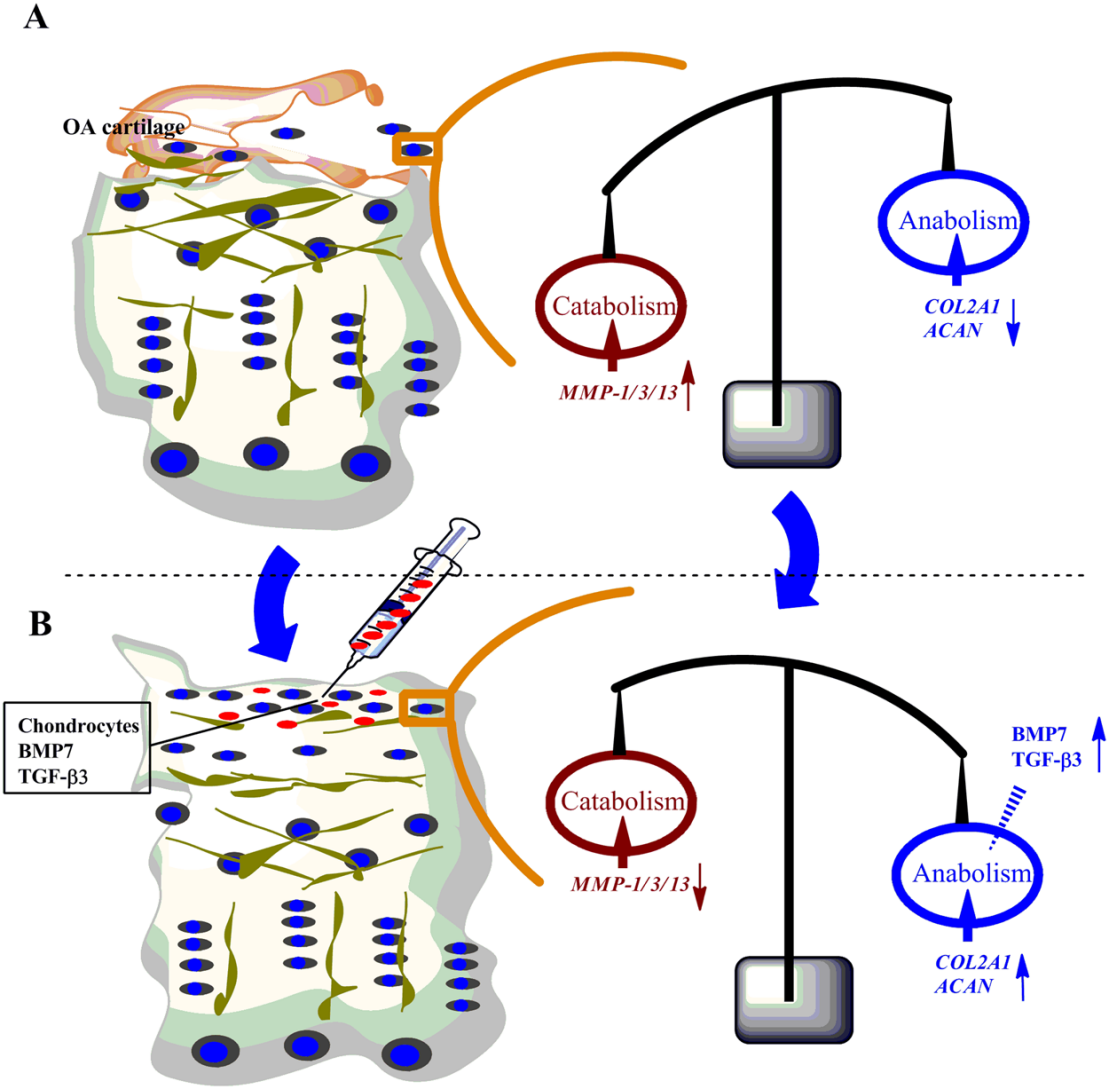
The schematic diagram of OA treatment by TGF-β3 and BMP7. (**A**) Osteoarthritic cartilage with a degenerative superficial layer and loss of chondrocytes shows higher catabolism, reflected by elevated *MMP-1/3/13* expression; (**B**) After treatment with the combination of TGF-β3 and BMP7, the superficial layer with chondrocytes with newly gained energy may exhibit higher anabolism, reflected by increased expression of *COL2A1* and *ACAN*.

To the best of our knowledge, this is the first study using human OA chondrocyte to study the potential application of BMP7 and TGF-β in cartilage tissue regeneration in 3D-cultured pellets under hypoxia. As Fig. 6 shows, the combination of TGF-β3 and BMP7 might readjust the balance between catabolism and anabolism by upregulating anabolic activities and downregulating catabolic activities in the treatment of osteoarthritic cartilage. Taken together, the combined treatment of BMP7 and TGF-β3 was superior in chondrocyte redifferentiation compared to single treatments both in normoxia and hypoxia. This study also indicate that a balanced combination of cytokines or growth factors in combination with an appropriate oxygen tension is required for efficient chondrocyte redifferentiation in pellet cultures.

## Electronic supplementary material

Supplemental Figures
