# Supplementary material for: Co-treatment of TGF-β3 and BMP7 is superior in stimulating chondrocyte redifferentiation in both hypoxia and normoxia compared to single treatments
Source: Sci Rep. 2018 Jul 6;8:10251. doi: 10.1038/s41598-018-27602-y (PMC6035177; doi:10.1038/s41598-018-27602-y)
Supplement: Supplementary file 1 — Supplemental Figures [file 41598_2018_27602_MOESM1_ESM.docx]

**Co-treatment of TGF-β3 and BMP7 is superior in stimulating chondrocyte redifferentiation in both hypoxia and normoxia compared to single treatments**

Xiaobin Huang, Leilei Zhong, Janine N. Post and Marcel Karperien*

Developmental BioEngineering, MIRA Institute for Biomedical Technology and Technical Medicine, University of Twente, Enschede 7500 AE, The Netherlands

* corresponding author: [h.b.j.karperien@utwente.nl](mailto:h.b.j.karperien@utwente.nl)

**Supplemental Figure 1. The protein expression of BMP7 was detected by IHC in each donor (scale bar=500 μm).** Images were taken using the Nanozoomer. G0, G1, G2, G3, G4, G5 = Grade 0, Grade 1, Grade 2, Grade 3, Grade 4, Grade 5 osteoarthritic cartilage.


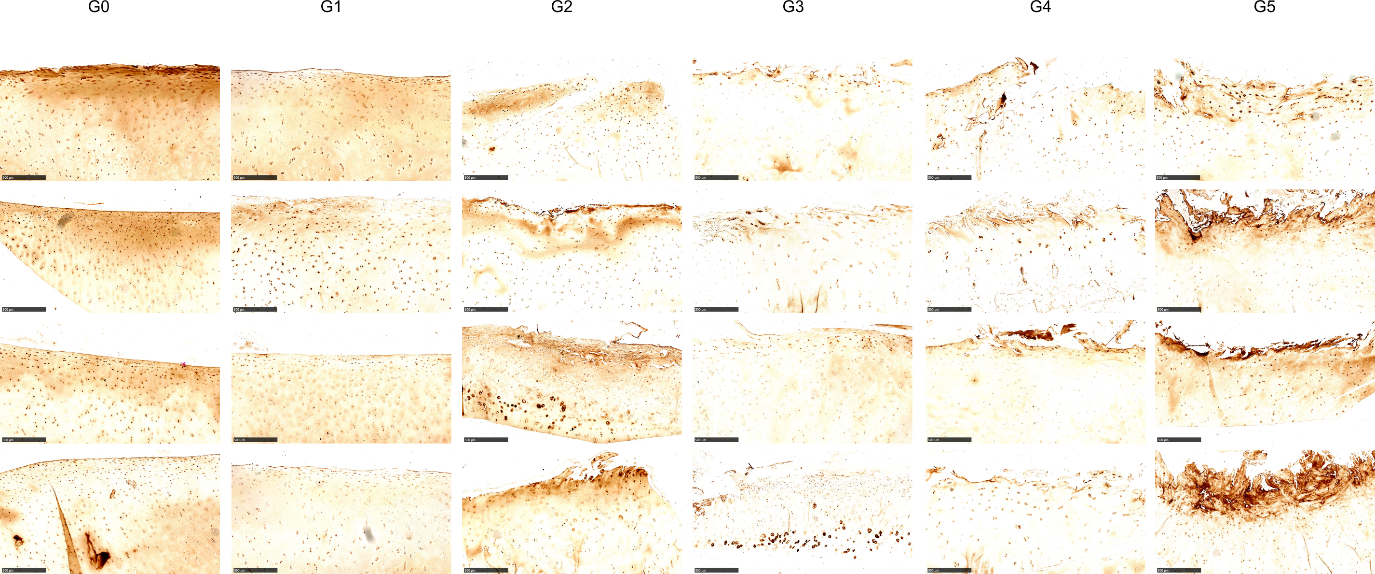


**Supplemental Figure 2. The protein expression of SMAD 1/5/8 was detected by IHC in each donor (scale bar=500 μm)**. Images were taken using the Nanozoomer. G0, G1, G2, G3, G4, G5 = OARSI Grade 0, Grade 1, Grade 2, Grade 3, Grade 4, Grade 5 osteoarthritic cartilage.

**
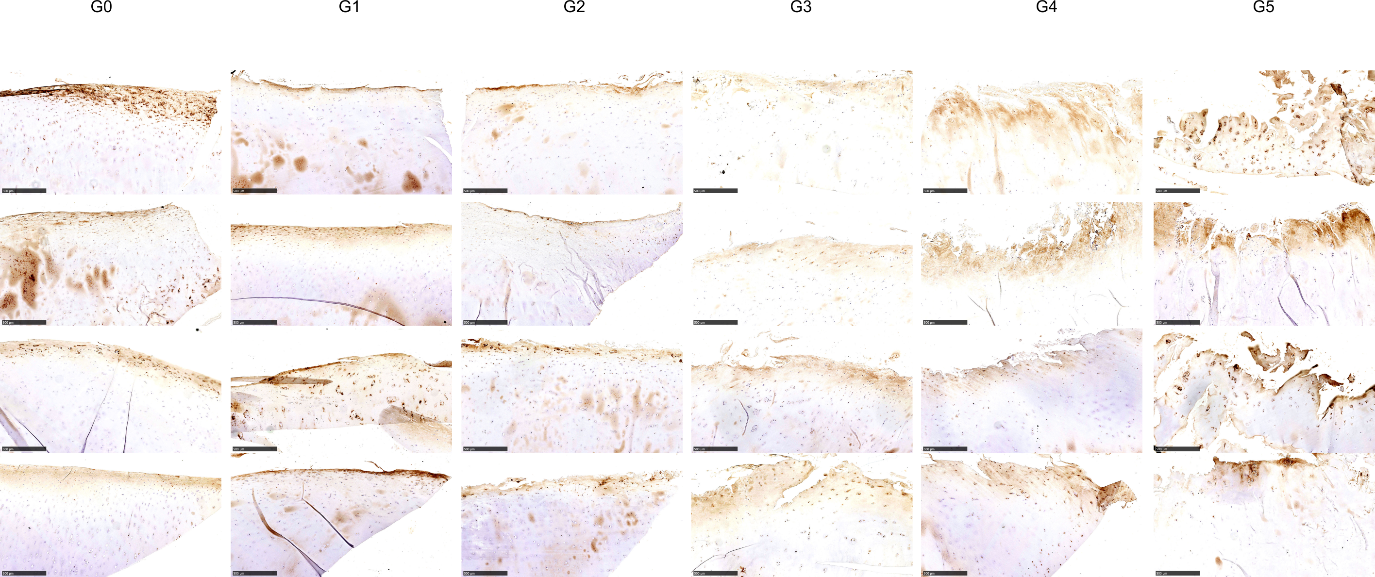
**

**Supplemental Figure 3. Measurement of type X collagen expression by immunofluorescence.** Type X collagen was detected by mouse anti- type X collagen antibody (red), followed by anti-mouse secondary antibody coupled to Alex 564. Fluorescent images were taken by BD pathway confocal microscopy. Cell nuclei were counterstained with DAPI. Scale bar=100μm. N, normoxia; H, hypoxia

**
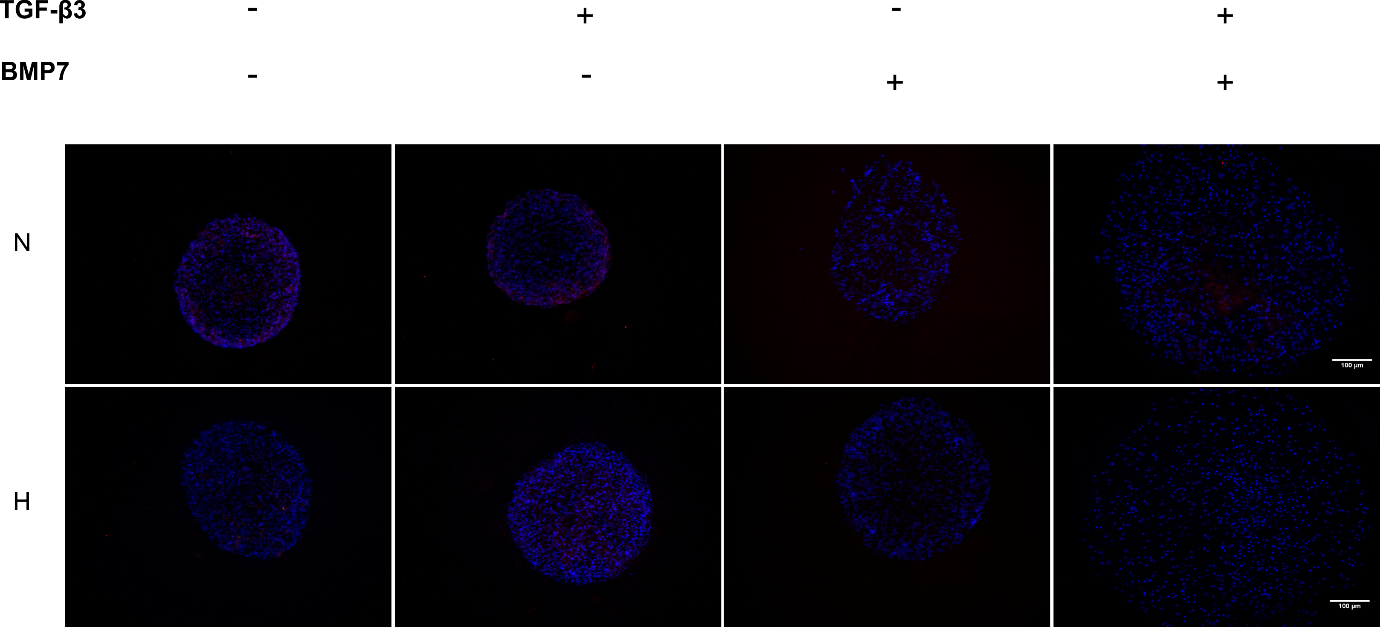
**
